# Oscillating U-Shaped Body for Underwater Piezoelectric Energy Harvester Power Optimization

**DOI:** 10.3390/mi10110737

**Published:** 2019-10-30

**Authors:** Iñigo Aramendia, Aitor Saenz-Aguirre, Ana Boyano, Unai Fernandez-Gamiz, Ekaitz Zulueta

**Affiliations:** 1 Nuclear Engineering and Fluid Mechanics Department, University of the Basque Country UPV/EHU, 01006 Vitoria-Gasteiz, Spain; inigo.aramendia@ehu.eus; 2 Automatic Control and System Engineering Department, University of the Basque Country UPV/EHU, 01006 Vitoria-Gasteiz, Spainekaitz.zulueta@ehu.eus (E.Z.); 3 Mechanical Engineering Department, University of the Basque Country UPV/EHU, 01006 Vitoria-Gasteiz, Spain; ana.boyano@ehu.eus

**Keywords:** energy harvesting, piezoelectric, pipelines, underwater networks, wireless sensor networks, control algorithm

## Abstract

Vibration energy harvesting (VeH) techniques by means of intentionally designed mechanisms have been used in the last decade for frequency bandwidth improvement under excitation for adequately high-vibration amplitudes. Oil, gas, and water are vital resources that are usually transported by extensive pipe networks. Therefore, wireless self-powered sensors are a sustainable choice to monitor in-pipe system applications. The mechanism, which is intended for water pipes with diameters of 2–5 inches, contains a piezoelectric beam assembled to the oscillating body. A novel U-shaped geometry of an underwater energy harvester has been designed and implemented. Then, the results have been compared with the traditional circular cylinder shape. At first, a numerical study has been carried at Reynolds numbers Re = 3000, 6000, 9000, and 12,000 in order to capture as much as kinetic energy from the water flow. Consequently, unsteady Reynolds Averaged Navier–Stokes (URANS)-based simulations are carried out to investigate the dynamic forces under different conditions. In addition, an Adaptive Differential Evolution (JADE) multivariable optimization algorithm has been implemented for the optimal design of the harvester and the maximization of the power extracted from it. The results show that the U-shaped geometry can extract more power from the kinetic energy of the fluid than the traditional circular cylinder harvester under the same conditions.

## 1. Introduction

Due to industrial development and the improved quality of human life, the economic and social demand for energy is growing. In recent years, the problems arising from the use of coal, petroleum, and other energy resources with high carbon content are becoming almost unbearable. The trend of annual average of atmospheric CO_2_ concentration has been increasing non-stop in the last decades. The global average surface temperature was reported to be approximately 1 °C higher than that of the preindustrial period [1]. The transition to an energy system that relies primarily on renewable energy sources has become one of the greatest challenges for alleviating climate change [2,3]. Clean energy sources such as wind, solar, geothermal, biomass, biofuels, waves, tidal, and hydropower can replace fossil fuels. With that in mind, water value is due not only to its agricultural and domestic use, but also to the possibility of being a source of energy, such as hydropower [4,5], ocean energy [6,7], or the energy obtained from its distribution network, for instance [8,9].

In this context, collecting small amounts of energy from the ambient in order to supply power to wireless devices has been investigated for the last decades. Moreover, in some cases where batteries are impractical, such as inaccessible remote systems, health monitoring, or body sensors, energy harvesting technology is very promising [10]. In fact, the power supply is one of the biggest challenges regarding the wireless sensor network applications. Frequently, the lifetime is confined to a battery supply, which is awkward [11]. Several environmental resources that offer enough power can be studied such as vibration, solar irradiation, thermal differences, and hybrid energy sources. Vibration energy harvesting (VeH) transforms mechanical energy obtained from ambient sources to electricity in order to power remote sensors. VeH technologies have been widely studied over the past decade [12,13,14]. Izadgoshasb et al. [15] proposed a new double pendulum-based piezoelectric system to harvest energy from human movements. They found a high increase in maximum output voltage in comparison to the conventional system and to an analogous system with only one pendulum. Furthermore, they stated that the double pendulum design could be further improved by varying some design parameters regarding dimensions or material. In a more recent work, Izadgoshasb et al. [16] proposed a multi-resonant harvester that consists of a cantilever beam with two triangular branches. They performed a parametric study using the finite element method (FEM) for the design optimization in order to get close resonances at low-frequency spectrum. Experimental results showed that the proposed harvester can harvest broadband energy from ambient vibration sources and is better than analogous piezoelectric energy harvesters with cantilever beams.

Energy harvesting from flow-induced vibrations has also attracted attention in the past years, and energy obtained from fluid–structure interaction (FSI) in different fluids, air, or liquid have been investigated by Elahi et al. [17]. Under flow loads, a structure can suffer different effects, such as limit cycle oscillations, chaotic movements, or internal resonances. From the point of view of aerodynamics, vortex-induced vibrations or vibrations caused by flutter or galloping can occur. Using piezoelectric devices placed in a flow field can convert large oscillations into electrical energy. An airfoil section placed on the end of cantilever piezoelectric beam can be used as a flutter energy harvester. Flutter speed is a critical value, from which the aerodynamic system becomes unstable. The self-excited oscillations that appear on the aeroelastic system once the critical value is overpassed are quite beneficial from the dynamic point of view; see Abdelkefi et al. [18]. Elahi et al. [19] modeled a nonlinear piezoelectric aeroelastic energy harvester that works on a postcritical aerolastic regime. An analytical model was developed taking into account the fluid–structure interaction and electromechanical performance. They concluded that higher electromechanical factor gives better harvesting. Wang et al. [20] conducted a study on a galloping-based piezoelectric energy harvester using isosceles triangle sectioned bluff bodies and applying computational fluid dynamics (CFD) to simulate the aerodynamic forces. They performed a parametric study in order to get the optimum vertex angle of the triangle and provided a guideline for efficient design. They determined that an angle of 130 degrees was the most adequate within the specific electromechanical coupling of their prototype. Dai et al. [21] investigated energy harvesting obtained from wind flow-induced vibrations. They compared experimentally four different test cases of piezoelectric energy harvesters, and based on the results they concluded which was the best orientation of the bluff body to design efficient devices. Jia et al. [22] presented an upright piezoelectric energy harvester (UPEH) that has a cylinder extension along its length. In the case with low speed wind, energy is obtained by vortex-induced vibrations (VIVs) that produce bending deformation. The UPEH can generate energy from low-speed wind by bending deformation produced by vortex-induced vibrations (VIVs). Zulueta et al. [23] developed a new control law for a contactless piezoelectric wind energy harvester, and afterwards, Bouzelata et al. [24] improved this wind energy harvester as a battery charger The simulation results proved that the device could power the battery when the wind speed is v = 2.7 m/s, taking into account that usually the moderate wind speed is considered to be 4 m/s.

Wang and Ko [25] developed a new piezoelectric energy harvester that converted flow energy into electrical energy by means of the oscillation of a piezoelectric film. They concluded that the obtained voltages based on the finite element model they proposed agree adequately with the experiment performed with various pressure differences in the pressure chamber. Therefore, they could use the model to predict the performance of the device, in terms of dimensions, material properties, and pressure loads. Silva-Leon et al. [26] proposed a novel approach to collect wind and solar energy at the same time. After extensive experiments, they founded out that their device was able to generate up to 3–4 mW of total power, which is enough to power remote sensors and small-scale portable electronics. Zhong et al. [27] showed that a type of graphene nanogenerator could generate electricity from the flow of different types of liquid, including water. Qureshi et al. [28] developed an analytical model of a novel and scalable piezoelectric energy harvester, where the kinetic energy from water flow-induced vibration was collected by means of piezoceramic cantilevers. They validated the model by means of a finite element simulation. They concluded that it is possible to install an energy harvester into the Turkey–Cyprus water pipeline with the capability to meet the power requirements of a wireless sensor node to monitor critical parameters.

Another way of collecting energy from water flow is the power generation from fluid flow in pipelines. This energy can be used to power wireless sensors networks; therefore, continuous monitoring of water quality and hydraulic parameters can be performed [29]. In addition, significant leakages can be detected in near real time [30]. Hoffmann et al. [31] presented a radial-flux energy harvester that obtained energy from water flow in water pipelines. Experimental results showed that the obtained energy could be used for powering smart water meter systems when the flow rate was at least 5 L/min. They pointed out that to achieve a power output less dependent on the flow rate, a fluidic bypass could be used. Shan et al. [32] presented a new underwater double piezoelectric energy harvesters system that consists of two harvesters placed in series with same parameters. The results of the experimental work showed that the performance of the double harvester can be improved by equaling the water speed, the specific gravity of the cylinder, and the spacing distance between the two harvesters.

The major novelty of the current research is the development of an innovative U-shaped geometry to be used as the oscillating body in an underwater energy harvester with the aim to increase the power output by the device. Three different geometries for the oscillating body were previously studied by Yao et al. [33]: Circular, triangular, and square. However, they concluded that the circular shape provided the best performance in terms of power output generation. Therefore, we have hypothesized that the proposed U-shaped geometry could improve the power generated by the underwater piezoelectric system. In the current work, we have implemented an Adaptive Differential Evolution (DE)-based (JADE) algorithm for the optimization of the design process of the harvester. JADE is an optimization algorithm based on evolutionary principles that is intended to find the maximum/minimum values of a cost function. In this analysis, a multivariable JADE algorithm intended to maximize the power extracted from the harvester has been designed. The two parameters optimized with the JADE algorithm are the structural spring of the harvester and the constant gain associated to its control algorithm.

## 2. Differential Evolution-Based Optimization

### 2.1. Differential Evolution Algorithm

The Differential Evolution (DE) algorithm, first introduced by Storn et al. [34], is an optimization technique based on evolutionary principles. As stated in [34], three main concepts are required for the correct performance of an optimization algorithm such as the DE: The ability to find the global optimum and not get stuck in local optimums, the fast convergence, and simplicity in the configuration parameters.

**Figure 1:**
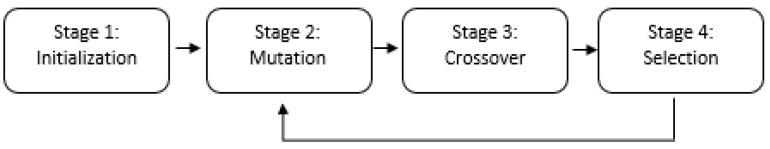
Pipeline of the operating principle of a Differential Evolution (DE) optimization algorithm.

As it is shown the pipeline in Figure 1, in order to fulfill the previously listed requirements, the DE algorithm proposes the execution of an initial Initialization stage, and the iterative execution of three main steps: Mutation, Crossover, and Selection.

The basic operation of a conventional DE algorithm is explained in detail in the work of Zhang et al. [35]. The first step is the generation (normally at a random process inside certain limit values) of the initial population of the algorithm; see Equation (1). To that purpose, the number of independent variables *N* and the size of the initial population *P* must be defined:

(1) $$x_{0}=\left\{x_{i,k,0}\right\}$$

where i=1:1:N and k=1:1:P.

After the initialization, the proposed iterative process is an emulation of the natural evolution. First, some individuals suffer from mutations that can improve/worsen their survival capabilities. To that end, different mutation strategies can be implemented in the DE algorithm. One of the most widely used mutation strategies, known as *“DE/randl1”*, is shown in Equation (2):

(2) $$v_{i,G}=x_{r1,G}+F_{i}\cdot \left(x_{r2,G}-x_{r3,G}\right)$$

Next, in the crossover or recombination step, the characteristics of two different individuals are combined to form a descendant individual, as it is shown in Equation (3):

(3) $$u_{j,i,G}=\left\{\begin{array}{c} if\;rand\left(0,1\right)\leq CR_{i}\;or\;j=j_{\mathrm{rand}}\;\;\;\;\;\;then\;\;\;\;\;u_{j,i,G}\\ else\;\;\;\;\;\;\;\;\;\;\;\;\;\;\;\;\;\;\;\;\;\;\;\;\;\;\;\;\;\;\;\;\;\;\;\;\;\;\;\;\;\;\;\;\;\;\;\;\;\;\;\;\;\;\;\;\;\;\;\;\;\;\;\;\;\;\;\;\;x_{j,i,G} \end{array}\right\}$$

where $j_{\mathrm{rand}}\in \left(1,N\right)$ is defined randomly at each iteration and $CR_{i}$ is the crossover probability, which is constant in the conventional DE algorithm.

Finally, the fitness of the individuals of the new generation is compared to the value of the fitness of the previous generation, and the best individuals are selected.

(4) $$u_{i,g+1}=\left\{\begin{array}{c} if\;f\left(u_{j,i,G}\right)<f\left(x_{j,i,G}\right)\;\;\;\;\;\;then\;\;\;\;\;\;\;\;\;\;\;\;\;\;\;\;\;\;\;\;\;u_{j,i,G}\\ else\;\;\;\;\;\;\;\;\;\;\;\;\;\;\;x_{j,i,G} \end{array}\right\}$$

This process is repeated during a defined number of generations, and after some iterations the individuals with the best characteristics remain and the evolutionary algorithm converges toward an optimal result.

As stated by Zhang et al. [36], DE algorithms have been widely used due to their simplicity, small number of configuration parameters, and good performance in optimization cases. Nevertheless, one of the problems present in these algorithms is the difficulty associated to the adequate setting of the configuration parameters. As explained in the work of Zhang et al. [35], both theoretical and experimental studies have been proposed for the setting of these parameters (especially the mutational factor *F* and the crossover probability *CR*). Nevertheless, the absence of clear guidelines and the necessity for trial and error tuning cause difficulties in achieving a good performance of the algorithm. In order to improve the results of DE algorithms, many variants have been developed and proposed in the literature: Self-adaptive Differential Evolution (SaDE), Self-adaptive Differential Evolution with Neighborhood Search (SaNSDE), Self-adaptive Differential Evolution (jDE), Differential Evolution with Global and Local neighborhoods (DEGL), Adaptive Differential Evolution (JADE), Composite Differential Evolution (CoDE), and Improved Adaptive Differential Evolution (IJADE).

### 2.2. JADE: Adaptive Differential Evolution

The JADE algorithm is a variation of the DE that belongs to the group of the adaptive parameter control algorithms, as introduced by Zhang et al. [35]. This means that the adaption of the configuration parameters is carried out according to the status of the search process of the algorithm. Some additional examples of adaptive parameter control algorithms are SaDE, jDE, and SaNSDE.

The JADE algorithm was first introduced in the work of Zhang et al. [37]. Later, a second version of the algorithm was presented by Zhang et al. [35]. The objective of the modifications introduced in the JADE strategy with respect to the DE algorithm is the improvement of the convergence of the algorithm and the diversification of the population that is used through the execution of it.

According to the work of Zhang et al. [37], the JADE algorithm is considered to improve the performance of the conventional DE algorithms by implementing a novel mutation strategy referred as “*DE/current-to-p best*” and a new method for the adaption of the mutational factor *F* and the crossover probability *CR*. The recombination and selection steps of the JADE algorithm are the same of the DE algorithm, which were presented in Equations (3) and (4), respectively.

The “*DE/current-to-p best*” mutation method introduced in the JADE algorithm can be expressed as shown in Equation (5):

(5) $$v_{i,G}=x_{i,G}+F_{i}\cdot \left(x_{best,G}^{p}-x_{i,G}\right)+F_{i}\cdot \left(x_{r2,G}-x_{r3,G}\right)$$

where *p* is the per one value of the number of the best individuals selected for the mutation.

The adaption at each generation of the crossover probability *CRi* is carried out by random generation according to a normal distribution and a predefined value of a standard deviation, as it is shown in Equation (6):

(6) $$CR_{i}=randn_{i}\left(\mu_{CR}\right)$$

Similarly, the adaption at each generation of the mutation factor *Fi* is carried out by random generation according to a Cauchy distribution with predefined parameter values, as shown in Equation (7):

(7) $$F_{i}=randn_{i}\left(\mu_{F}\right)$$

According to Islam et al. [38], with the use of the JADE algorithm, a diversity of the population, which avoids a premature convergence of the optimization algorithm, and a more reliable performance of the algorithm are achieved.

## 3. Harvester Description

**Figure 2:**
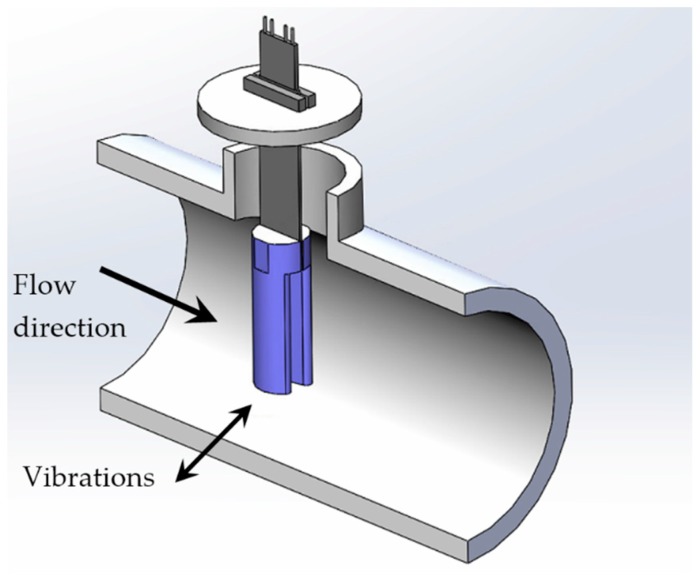
Energy harvester assembled inside a water pipe with the U-shaped geometry as the oscillating body (not to scale).

The mechanism presented in the current study is connected with the work of Cottone et al. [39] and on the computational fluid dynamics simulations of Aramendia et al. [40]. This device, which is intended to be used inside water pipelines of 2–5 inches of diameter, contains a piezoelectric beam assembled to an oscillating body, as shown in Figure 2.

**Figure 3:**
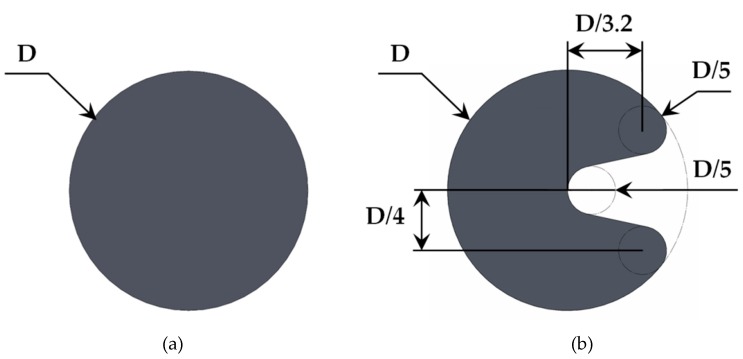
Geometries for the oscillating body. (**a**) Circular cylinder and (**b**) U-shaped geometry.

The impact of the water results in vibrations because of the vortices generated in the region closely behind the oscillating body. Additionally to the cylinder geometry, an innovative U-shaped geometry has been proposed as the oscillating body to optimize the extraction of kinetic energy from the water incoming through the water pipe. Figure 3 shows the dimensions of both geometries used in the present study.

## 4. Computational Setup

This section is devoted to provide a detailed description of the numerical model developed to characterize the operation of both oscillating bodies in an underwater harvester. The length of the computational domain consists of 40 times the body diameter (D) behind the oscillating body. This has been considered enough to study accurately the vortices generated by the water passing around the body. Diameters of *D* = 10 mm and *D* = 20 mm have been considered in this study for both geometries, as illustrated in Figure 3.

A velocity inlet and pressure outlet boundary condition has been defined as well as slip condition for the top and bottom boundaries. CFD tools require the subdivision of this computational domain into a number of smaller subdomains to solve the flow physics. Therefore, the mesh generation is a very relevant issue in the pre-process stage. The mesh carried out consists of 2D polyhedral cells; most of them are placed in the area behind the body after the definition of a fully anisotropic wake refinement. Additionally, a volumetric control has been designed to refine the mesh around the body and to keep a y+ value less than 1. The mesh dependency study of the previous work of Aramendia et al. [40] was used to verify the accuracy of the solution.

The Reynolds Averaged Navier–Stokes (RANS) equations have been applied to reach the numerical solution of the unsteady state flow involved. The finite volume method has been used to discretize the integral form of the conservation equations with the CFD commercial software STAR-CCM+ (v. 11.06.011, CD-adapco, Melville, NY, USA) [41]. An upwind scheme [42] was used to discretize the convective terms, ensuring the robustness of the solution. The eddy viscosity models (EVM) with two transport equations have been chosen to define the turbulence modeling by means of the k-ω shear stress transport (SST) turbulence model developed by Menter [43]. A time-step of 0.002 s and 15 inner iterations have been defined as the optimal configuration to capture the vortex shedding. A second-order temporal discretization has been used in all the simulations presented in the current study. The solution obtained with the simulations was considered converged when satisfactory residuals were achieved on pressure, turbulence, and velocity quantities.

## 5. Computational Results

Four Reynolds numbers have been chosen to study the cross flow (Re = 3000, 6000, 9000, and 12,000). The Re is a dimensionless number based on the oscillating body diameter (D) and was obtained by Equation (8), where *ρ*_water_ and *µ* correspond to the density and dynamic viscosity of water at a temperature of 15 °C:

(8) Re = Vwater × D × ρwaterμ

The water velocity V_water_ at the inlet has been changed to achieve the different Re numbers. The numerical solution in all cases was simulated for a period of time of 20 seconds. Table A1 and Table A2 of the Appendix A show the contour lines of the vorticity at t = 20 s for each Re number studied (Re = 3000, 6000, 9000, and 12,000) and for each geometry proposed as the oscillating body. The oscillating vortex pattern, convection, and diffusion of the vortices are visible. The body lift is calculated by means of CFD tools.

**Figure 4:**
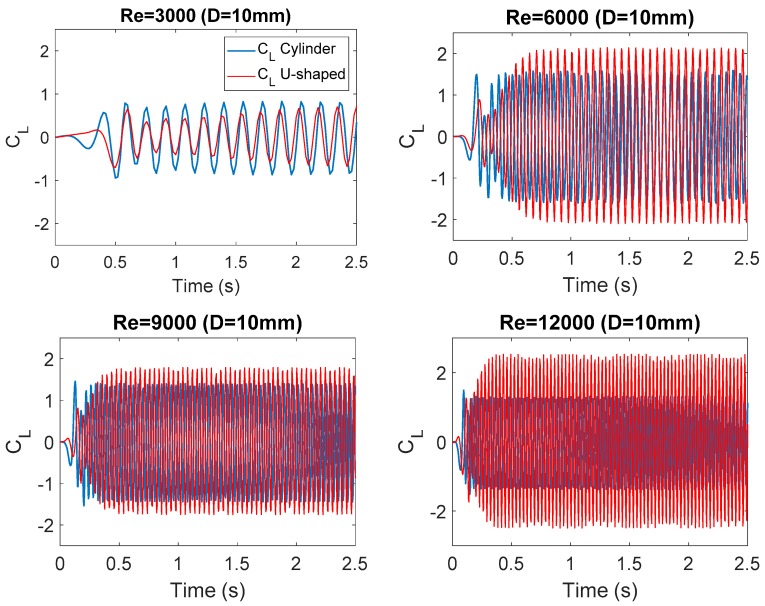
Evolution of the lift coefficient at each Reynolds number (*D* = 10 mm).

**Figure 5:**
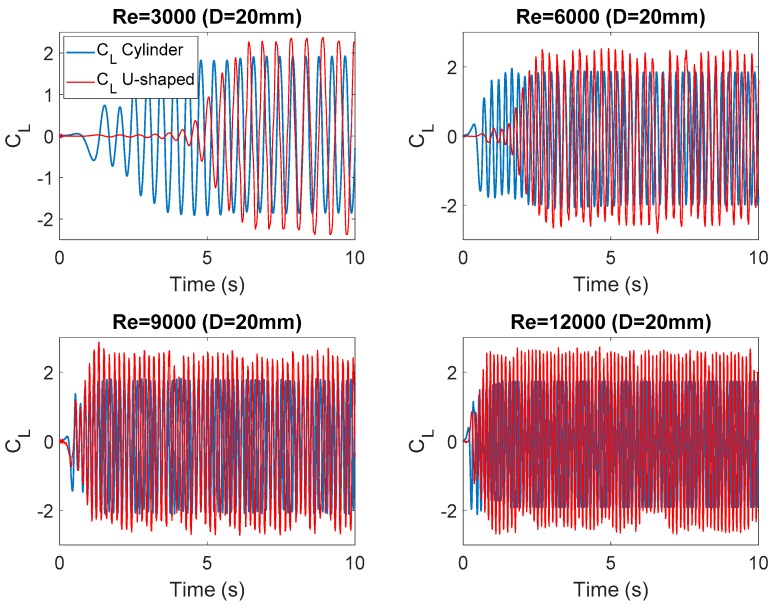
Evolution of the lift coefficient at each Reynolds number (*D* = 20 mm).

Figure 4 and Figure 5 show the evolution of the lift coefficient C_L_ at each Re number investigated. This dimensionless coefficient is calculated by Equation (9), where F_L_ represents the force perpendicular to the flow direction caused by the water in the oscillating body, i.e., the circular cylinder and the U-shaped geometry, respectively.

(9) $$C_{L}\;=\;\frac{F_{L}}{0.5{\;\times \;\rho }_{\mathrm{water}}\;\times \;U_{\infty }^{2}\;\times \;D}$$

## 6. JADE-Based Underwater Piezoelectric Energy Harvester Optimization

As it was explained in Section 3, a new geometry for an underwater piezoelectric energy harvester is presented in this document. The proposed novel geometry is considered to improve the performance of cylindrical devices used in conventional energy harvesting systems.

A detailed model of the dynamics of an underwater piezoelectric energy harvester system is presented in the work of Aramendia et al. [40]. The selection of the optimal parameters during the design process of the harvester enables maximizing the power generated by the harvesting system. The extracted power has been calculated by means of Equation (10):

(10) $$P\;=\;{\left(\frac{\alpha \cdot a}{K_{\mathrm{trans}}}\right)}^{2}\frac{{\overset{\cdot }{\theta }}^{2}}{K_{p}}={\left(\frac{\alpha \cdot a}{K_{\mathrm{trans}}}\right)}^{2}\frac{1}{K_{p}}{\left(\frac{a_{1}w_{0}{\cdot C}_{L\cdot \max}}{\sqrt{{\left(a_{4}{-a}_{2}w_{0}{}^{2}\right)}^{2}+{\left(a_{3}w_{0}\right)}^{2}}}\sin\left(w_{0}t\right)\right)}^{2}$$

Thus, the mean value of the instantaneous power is determined over the period given by an angular pulsation of the lift coefficient $w_{0}$; see Equation (11):

(11) $$P_{\mathrm{mean}}={\left(\frac{\alpha \cdot a}{K_{\mathrm{trans}}}\right)}^{2}\frac{1}{2{\cdot K}_{p}}{\left(\frac{a_{1}w_{0}{\cdot C}_{L\cdot \max}}{\sqrt{{\left(a_{4}{-a}_{2}w_{0}{}^{2}\right)}^{2}+{\left(a_{3}w_{0}\right)}^{2}}}\right)}^{2}$$

Table 1 and Table 2 show the system model input parameters and variables used in the control of the energy harvester, respectively.

In this way, the value of the parameter Kspring and the value of the control parameter Kp stand as two crucial variables for the optimization of the harvester. The Kspring parameter refers to the value of the spring constant of the torsion spring introduced to the hydro-mechanical system of the harvester. The objective of the torsion spring is to maintain the vertical equilibrium of the harvester in the absence of piezoelectric forces. The expression that models the action of the torsion spring in the harvester system is given in Equation (12):

(12) $$J_{wt}\cdot \frac{d^{2}\theta }{d^{2}t}=T_{\mathrm{hydro}}-K_{\mathrm{Spring}}\cdot \theta -f\cdot \frac{d\theta }{dt}-T_{m}$$

The Kp parameter refers to the proportional gain of the control law proposed for the performance of the underwater piezoelectric energy harvester. The action of the control system of the harvester is given by the following expression in Equation (13):

(13) $$K_{P}\cdot V_{1}=\alpha \cdot \frac{du_{1}}{dt}-C\cdot \frac{dV_{1}}{dt}$$

Due to the all the existent possibilities, the optimal setting of both Kspring and Kp parameters could be complicated, which would result in an inefficient operation of the harvester. Consequently, in this paper, a JADE optimization algorithm has been developed and implemented in order to obtain the optimal values of the Kspring and Kp parameters and thus optimize the performance of the system.

The cost function selected for the JADE algorithm is the power generated by the harvester system calculated in Equation (11). The power generated by the harvester is dependent on both Kp and the Kspring parameters and could be expressed as in Equation (14):

(14) $$P=f\left(K_{P},K_{\mathrm{Spring}}\right)$$

The configuration parameters defined for execution of the JADE algorithm presented in this paper are listed in Table 3.

Different scenarios corresponding to various harvester geometries and different Reynolds numbers of the fluid actuating on the harvester, all of them already introduced in Section 3, have been considered and optimized with the application of the JADE algorithm in order to find the best combination of Kp and Kspring parameters that maximizes the energy production of the underwater piezoelectric energy harvester.

**Figure 6:**
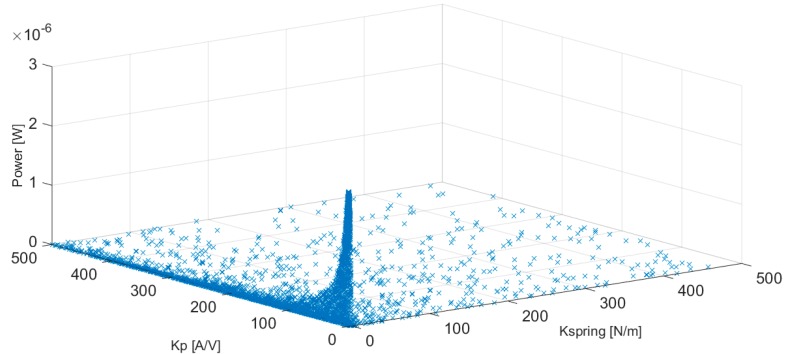
Progress of the JADE optimization algorithm. *D* = 10 mm circular cylinder-based harvester and Re = 3000.

An illustration of the progress of the JADE optimization algorithm corresponding to the cylindrical configuration of the harvester with a *D* = 10 mm and a Reynolds number equal to 3000 is presented in Figure 6.

As observed in Figure 6, the search of the optimization algorithm converges toward the optimum combination of the input parameters, Kp and Kspring, until the best solution that maximizes the power generated by the energy harvester is obtained.

## 7. Results

The search of the optimal combination of the Kp and Kspring parameter values for the maximization of the power generated by the harvester system through the implementation of a JADE algorithm has been proposed in Section 6. The results obtained for each one of the analyzed scenarios are represented in Table 4 and Table 5 for *D* = 10 mm and *D* = 20 mm, respectively. The last column of each table represents the increment of the power ΔPower (%) generated by the U-shape underwater energy harvester with respect to the traditional underwater harvester based on a cylindrical oscillating body.

**Figure 7:**
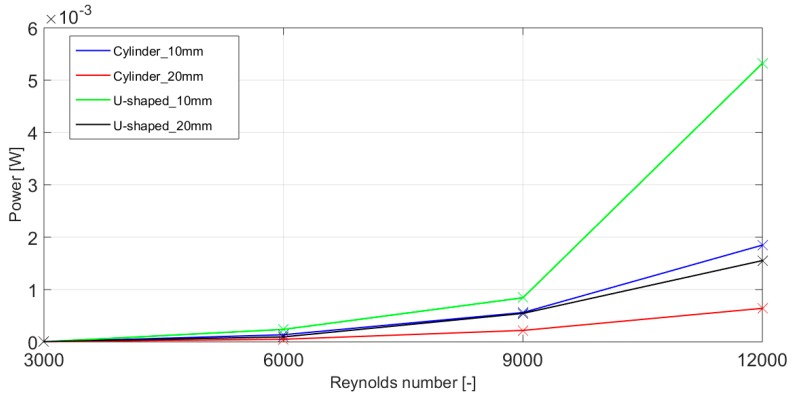
Comparison of the optimal power generated by the proposed four different energy harvesting system geometries.

The power generated by the harvesting system is considerably improved with the application of the proposed U-shaped geometry, especially for cases at higher Reynolds numbers, as observed in Table 4 and Table 5. In general, the power achieved by the U-shaped geometry is larger than the cylinder for both diameters considered. However, for the lowest Reynolds number studied, Re = 3000 and *D* = 10 mm, the power achieved by the U-shaped based harvester is lower than the one obtained by the cylinder. The largest power output is achieved at Re = 12,000 and *D* = 10 mm for both geometries. The cylinder oscillating body reaches a power of 1848.3 µW, and the U-shape geometry gets the maximum power with a value of 5321.7 µW, as shown in Table 4. Nevertheless, it must be taken into account that the water velocity associated at this high Reynolds number is difficult to obtain in the water pipes considered in this study from 2 to 5 inches of diameter. A graphical comparison of the optimal power generated by the analyzed four different harvester geometries for different Reynolds number values is presented in Figure 7. The present U-shaped harvester with an oscillating body size of *D* = 10 mm generates up to 5.2 mW at Re = 12,000. This result shows a significant improvement from the literature; please see the model presented on a review on mechanisms for piezoelectric-based energy harvesters for an underwater harvester [17], which is able to produce merely 0.9 mW at the same Re number.

**Figure 8:**
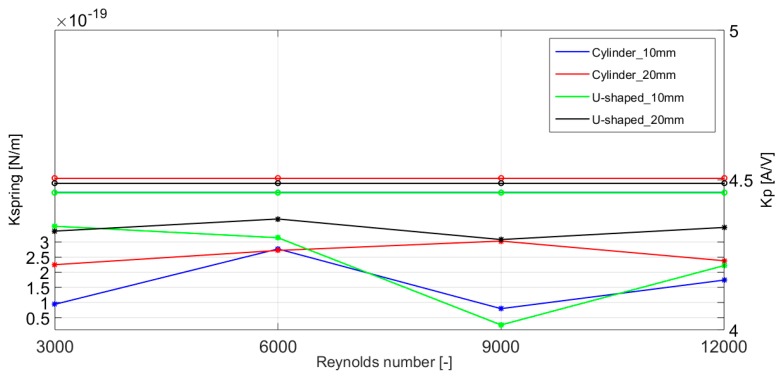
Comparison of the optimal values of the Kp and Kspring parameters for the proposed four different energy harvesting system geometries.

Similarly, a comparison of the optimal values of the Kp and Kspring parameters for each one of the four analyzed harvester geometries and for different Reynolds number values is presented in Figure 8.

There are slight differences in the optimal value of the Kp and Kspring parameters, especially for the geometries with *D* = 10 mm. This could be translated in a non-optimal performance of the system and in a reduction in the power generation of the harvesting system, with its subsequent decrease of energy yield. These increased power generation of the harvesting system and the differences in the optimal values of the Kp and Kspring parameters prove the correct performance of the proposed harvester geometry and the JADE optimization algorithm presented in this paper.

## 8. Conclusions

In the current work, a numerical study of an underwater piezoelectric energy harvester has been carried out for the extraction of kinetic energy from the water flow. The mechanism, which is planned to be used inside water pipelines of 2–5 inches of diameter, contains a piezoelectric beam assembled to an oscillating body. Two different geometries for the oscillating body have been considered. The first one is the traditional circular cylinder, and the second one is a novel U-shaped geometry. Both geometries have been studied for two different diameters: *D* = 10 and 20 mm. Thus, 2D numerical simulations have been performed around each proposed geometry at Reynolds numbers Re = 3000, 6000, 9000, and 12,000. Simulations in unsteady-state conditions were made during a period of time of 20 seconds in order to evaluate the vortex shedding generated in the region behind the oscillating bodies. The lift coefficient of the oscillating bodies obtained in the simulations has been used as an input variable in the control system.

Furthermore, a multivariable JADE-based optimization algorithm has been designed to optimize the design process of the harvester and maximize the power extracted from it. The two parameters optimized with the JADE algorithm are the structural spring of the harvester and the constant gain associated to its control algorithm. According to the obtained results, the power generated by the U-shape-based energy harvester is always larger than the one obtained by the circular cylinder for all the Reynolds numbers studied except for Re = 3000 and *D* = 10 mm. The maximum power extracted from the harvester is 5321.7 µW and corresponds to the case with Re = 12,000 and *D* = 10 mm. The results show that thanks to the U-shaped geometry of the oscillating body and to the JADE optimization algorithm, the power output of the harvester has significantly improved.

The power generated by the underwater piezoelectric energy harvester follows an exponential law for all the cases investigated, including the U-shaped geometry. Additionally, the proportional gain of the control law maintains approximately constant at the water speeds studied in the current work.

## Figures and Tables

**Table 1:** Model parameters.

Name	Definition	Value	Units
$\rho_{\mathrm{water}}$	Fluid density	997.5	kg/m^3^
*K*_trans_	Transduction gain	2	-
f	Frictional coefficient	0.01	(N·m·s)/rad
*a*	Force application distance point	0.01	m
*α*	Voltage induced bending factor	100	A s/m
*C*	Piezoelectric capacitance	1	nF

**Table 2:** Model variables.

Name	Definition	Units
*C*_*L*,max_	Maximum lift coefficient	-
*V*_1_	Piezoelectric voltage	V
*t*	Time	s
*θ*	Beam angle	rad
*K*spring	Spring constant	N/m
*Kp*	Proportional gain	A/V
*T_m_*	Moment generated by the piezoelectric	N·m
*T*_Hydro_	Hydro-mechanical torque	N·m
$\omega_{0}$	Angular pulsation of the lift coefficient	rad/s
*J_wt_*	Oscillating body inertia moment	Kg·m^2^
*u*_1_	Reference of the piezoelectric deflection	m

**Table 3:** Configuration parameters of the adaptive differential evolution (JADE) algorithm.

Explanation	Symbol	Value
Number of variables	N	2
Initial population size	P	200
Number of iterations	Niter	2000
Mutation ratio	F	Adaptive
Crossover probability	CR	Adaptive
Mutation ratio adaption parameter	$\mu_{F}$	0.5
Crossover probability adaption parameter	$\mu_{CR}$	0.5
Kp maximum value	Kpmax	500
Kp minimum value	Kpmin	0
Kspring maximum value	Kspringmax	500
Kspring maximum value	Kspringmin	0
“DE/current-to-p best” mutation parameter	p	0.1

**Table 4:** Results of the JADE optimization algorithm for the cylinder and U-shaped oscillating bodies with *D* = 10 mm.

	Cylinder *D* = 10 mm			U-shape *D* = 10 mm			
Re	Kspring	Kp	Power [µW]	Kspring	Kp	Power [µW]	ΔPower (%)
3000	9.41 × 10^−20^	4.4585	2.25	3.52 × 10^−19^	4.4574	1.75	–28.57
6000	2.77 × 10^−19^	4.4585	135.76	3.14 × 10^−19^	4.4574	237.8	42.91
9000	7.96 × 10^−20^	4.4585	560.16	2.58 × 10^−20^	4.4574	842.75	33.53
12,000	1.74 × 10^−19^	4.4585	1848.3	2.22 × 10^−19^	4.4574	5321.7	65.27

**Table 5:** Results of the JADE optimization algorithm for the cylinder and U-shaped oscillating bodies with *D* = 20 mm.

	Cylinder *D* = 20mm			U-shape *D* = 20mm			
Re	Kspring	Kp	Power [µW]	Kspring	Kp	Power [µW]	ΔPower (%)
3000	2.25 × 10^−19^	4.5054	3.03	3.36 × 10^−19^	4.4886	4.63	34.56
6000	2.72 × 10^−19^	4.5054	49.857	3.76 × 10^−19^	4.4886	94.54	47.26
9000	3.03 × 10^−19^	4.5054	218.59	3.08 × 10^−19^	4.4886	543.02	59.75
12,000	2.38 × 10^−19^	4.5054	640.74	3.48 × 10^−19^	4.4886	1553	58.74

## Appendix A

**Table A1:** Vortex shedding comparison behind the circular cylinder and the U-shaped geometry at different Reynolds numbers where *D* = 10 mm.

Re	Wake Development (Cylinder)	Wake Development (U-Shaped)
3000		
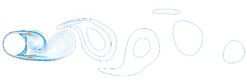
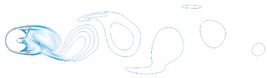
6000		
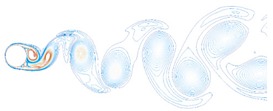
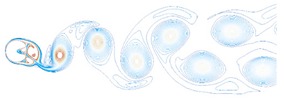
9000		
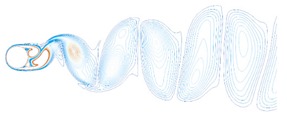
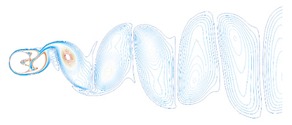
12,000		
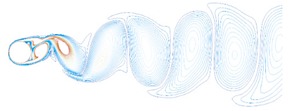
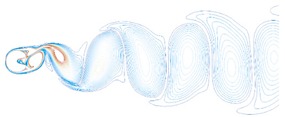

**Table A2:** Vortex shedding comparison behind the circular cylinder and the U-shaped geometry at different Reynolds numbers where *D* = 20 mm.

Re	Wake Development (Cylinder)	Wake Development (U-Shaped)
3000		
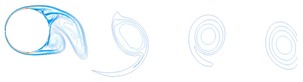
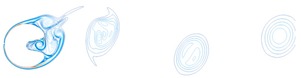
6000		
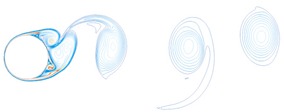
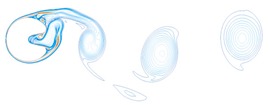
9000		
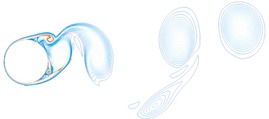
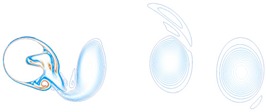
12,000		
